# mRNA Export from Mammalian Cell Nuclei Is Dependent on GANP

**DOI:** 10.1016/j.cub.2009.10.078

**Published:** 2010-01-12

**Authors:** Vihandha O. Wickramasinghe, Paul I.A. McMurtrie, Anthony D. Mills, Yoshinori Takei, Sue Penrhyn-Lowe, Yoko Amagase, Sarah Main, Jackie Marr, Murray Stewart, Ronald A. Laskey

**Affiliations:** 1 MRC Cancer Cell Unit, Hutchison/MRC Research Centre, Hills Road, Cambridge CB2 0XZ, UK; 2 MRC Laboratory of Molecular Biology, Hills Road, Cambridge CB2 0QH, UK

**Keywords:** RNA

## Abstract

Bulk nuclear export of messenger ribonucleoproteins (mRNPs) through nuclear pore complexes (NPCs) is mediated by NXF1. It binds mRNPs through adaptor proteins such as ALY [1, 2] and SR splicing factors [3] and mediates translocation through the central NPC transport channel via transient interactions with FG nucleoporins [4–10]. Here, we show that mammalian cells require GANP (germinal center-associated nuclear protein) for efficient mRNP nuclear export and for efficient recruitment of NXF1 to NPCs. Separate regions of GANP show local homology to FG nucleoporins, the yeast mRNA export factor Sac3p, and the mammalian MCM3 acetyltransferase. GANP interacts with both NXF1 and NPCs and partitions between NPCs and the nuclear interior. GANP depletion inhibits mRNA export, with retention of mRNPs and NXF1 in punctate foci within the nucleus. The GANP N-terminal region that contains FG motifs interacts with the NXF1 FG-binding domain. Overexpression of this GANP fragment leads to nuclear accumulation of both poly(A)^+^RNA and NXF1. Treatment with transcription inhibitors redistributes GANP from NPCs into foci throughout the nucleus. These results establish GANP as an integral component of the mammalian mRNA export machinery and suggest a model whereby GANP facilitates the transfer of NXF1-containing mRNPs to NPCs.

## Graphical abstract

**Figure:**
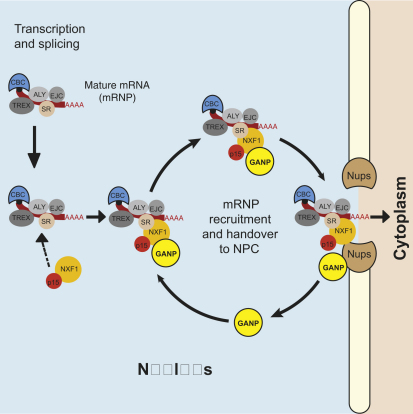

## Results and Discussion

Although the yeast mRNA export machinery is well characterized and is partly conserved in mammalian cells, there are important functional differences. First, the TREX (*tr*anscription-*ex*port) complex, which coordinates many steps in the gene expression pathway [11], is recruited cotranscriptionally in yeast [12] but by the splicing machinery in mammals [13, 14]. Second, although Yra1 is essential for mRNA export in yeast cells [15], the metazoan homolog ALY is dispensable [16]. This suggests that additional adaptor proteins mediate the interaction between the principal mRNA nuclear export factor, NXF1, and its cargoes in metazoa [16]. Moreover, some active genes in yeast, such as *GAL1*, become tethered to nuclear pore complexes (NPCs) in a process known as “gene gating” [8, 17]. In contrast, the majority of active genes in mammalian cells lie in transcription foci or “factories” deep within the nucleus [18]. These fundamental differences may reflect the much greater incidence of introns in metazoan genes. Here, we identify GANP (germinal center-associated nuclear protein) as an integral component of the mammalian mRNA export machinery. GANP is upregulated in germinal center B cells [19] and a variety of lymphomas [20]. However, these observations do not explain why GANP is expressed in essentially all mammalian cells, suggesting that it has a more general role. Confusion has arisen in databases and the literature because the *MCM3AP* (MCM3 acetylating protein) gene [21, 22] is contained entirely within the *GANP* gene. However, GANP residues 1–1259 have no counterpart in MCM3AP, and MCM3AP can be transcribed independently of GANP. We propose elsewhere that they should be referred to as independent but overlapping genes (V.O.W., P.I.A.M., A.D.M., Y.T., Y.A., S.M., J.M., and R.A.L., unpublished data).

**Figure 1:**
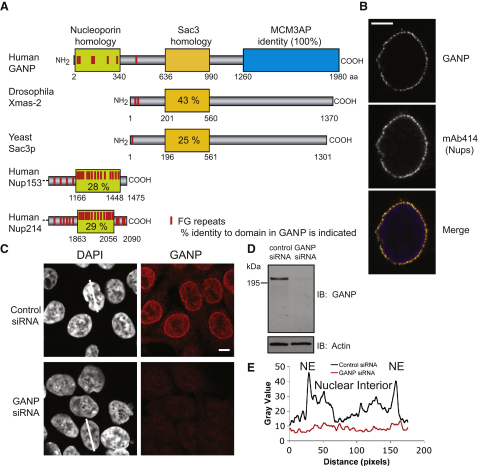
GANP Combines Features Found in Nucleoporins and Components of the mRNA Export Machinery and Is Partitioned between Nuclear Pore Complexes and the Nuclear Interior (A) GANP (germinal center-associated nuclear protein) protein sequence compared to *Drosophila* Xmas-2, yeast Sac3p, and human Nups 153 and 214. Red bars indicate FG repeats typical of many nucleoporins. Percent identities to domains in GANP are indicated. (B) Immunofluorescence with anti-GANP (top) shows that it colocalizes with FG-repeat nucleoporins (stained with mAb414, middle) in SKOV-3 nuclei isolated with Triton X-100. Merged image with DAPI nuclear staining is shown at bottom (blue). (C) Immunofluorescence with anti-GANP on intact HCT116 cells shows that GANP is located at nuclear pore complexes (NPCs) and in the nucleus. Immunofluorescence showed negligible staining of small interfering RNA (siRNA) GANP-depleted HCT116 cells. Nuclei are indicated by DAPI staining. Scale bar represents 5 μm. Identical microscope settings were used to acquire each pair of images. (D) HCT116 cells were depleted of endogenous GANP as above and analyzed by immunoblotting for GANP and actin (loading control). Control cells were transfected with a siRNA differing from GANP siRNA by two bases. (E) Scanning analysis of GANP intensity in control siRNA-treated and GANP-depleted cells with ImageJ software. Nuclei used for scanning and the scanning axis are indicated by white lines. Pairs of nuclei of same scan width as determined by DAPI staining were used. Nuclear envelope (NE) and nuclear interior are indicated.

GANP contains regions of homology to two classes of protein involved in nuclear trafficking (Figure 1A). Residues 1–340 show 23%–32% identity to regions of several highly conserved NPC proteins (FG nucleoporins) including a cluster of six FG motifs [23] (Figure 1A; see also Figure S1A available online), whereas residues 636–990 show 25% identity to Sac3p, a component of the yeast mRNA export machinery [23], and to *Drosophila* Xmas-2 (43% identity) [24] (Figure 1A; Figures S1B and S1C). However, the Sac3 homology domain represents only 18% of GANP, and it is present in other proteins that are not involved in mRNA export.

Immunoblotting with sheep antibodies raised against a unique region of GANP (residues 1050–1250) that is absent from MCM3AP recognized a 210 kDa band, which was abolished following small interfering RNA (siRNA) depletion (Figure 1D). Confocal immunofluorescence of intact HCT116 cells showed strong nuclear envelope staining and weaker nuclear interior staining. Both were abrogated following siRNA-mediated depletion of GANP (Figures 1C and 1E). Immunofluorescence of permeabilized human nuclei (Figure 1B) confirmed that this nuclear envelope staining colocalized with antibody mAb414 that recognizes four integral NPC components (Nups 62, 153, 214, and 358). Antibody access experiments showed that GANP is localized to the nuclear face of NPCs but is absent from the cytoplasmic face (Figure S1E).

**Figure 2:**
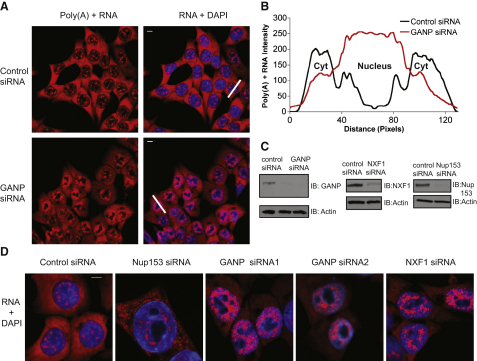
GANP Depletion Results in Nuclear Accumulation of poly(A)^+^RNA (A) Fluorescence in situ hybridization (FISH) showing nuclear accumulation of poly(A)^+^RNA in GANP-depleted cells 72 hr posttransfection. Merged image is shown in right panels. Scale bar represents 5 μm. (B) Scanning analysis of poly(A)^+^RNA intensity in control siRNA-treated and GANP-depleted cells. Pairs of cells with nuclei of same scan width as determined by DAPI staining were used for scanning, and the scanning axes are indicated by white lines. Scanning axis was chosen so as to bypass nucleoli in which poly(A)^+^RNA staining is not detected. Cytoplasm (Cyt) and nucleus are indicated. (C) Immunoblotting analysis of HCT116 cells depleted of Nup153, NXF1, and GANP. (D) FISH showing that poly(A)^+^RNA accumulates in punctate nuclear foci in GANP- and NXF1-depleted cells, but not in Nup153-depleted HCT116 cells 72 hr posttransfection. Poly(A)^+^RNA was identified with a Cy3-labeled oligo(dT) probe. Nuclei were stained with DAPI.

To ask whether GANP functions in mammalian mRNA export, we examined the effect of GANP depletion on poly(A)^+^RNA export via RNA fluorescence in situ hybridization (FISH). Nuclear accumulation of poly(A)^+^RNA was observed with two independent siRNAs directed against the unique region of GANP, but not with control siRNA that differed by two bases from that used to deplete GANP (Figures 2A and 2D). In control cells, most poly(A)^+^RNA was cytoplasmic, except for a few discrete foci in nuclei (Figure 2A), as observed previously [25]. In contrast, GANP depletion caused nuclear accumulation of poly(A)^+^RNA (Figures 2A and 2B), and mean nuclear polyA(+)RNA levels were ∼50% higher in GANP-depleted cells compared to control cells (97 versus 63), even without correction for the large unstained nucleolar volume (Figure S2A). Because the siRNA used corresponded to the unique region of GANP, the effects on mRNA export were specific for depletion of GANP and not MCM3AP. Importantly, nuclear import and CRM1-dependent export of STAT2 [26] proceeded in the absence of GANP, indicating that NPCs were functional for bidirectional transport of receptor-cargo complexes in these cells (Figure S2B). Thus, nuclear export of poly(A)^+^RNA is severely inhibited by GANP depletion.

Because GANP contains local homology both to nucleoporins and to Sac3p, we compared its depletion phenotype to those of either Nup153 or mRNA export factor NXF1. Figures 2C and 2D show that the punctate accumulation of poly(A)^+^RNA in the nucleus following GANP depletion closely resembles that seen following NXF1 depletion but differs from the pattern observed following Nup153 depletion. Poly(A)^+^RNA did not accumulate at the nuclear envelope of GANP-depleted cells but instead accumulated in a distinct punctate focal pattern throughout the nucleus, excluding nucleoli (Figure 2D).

**Figure 3:**
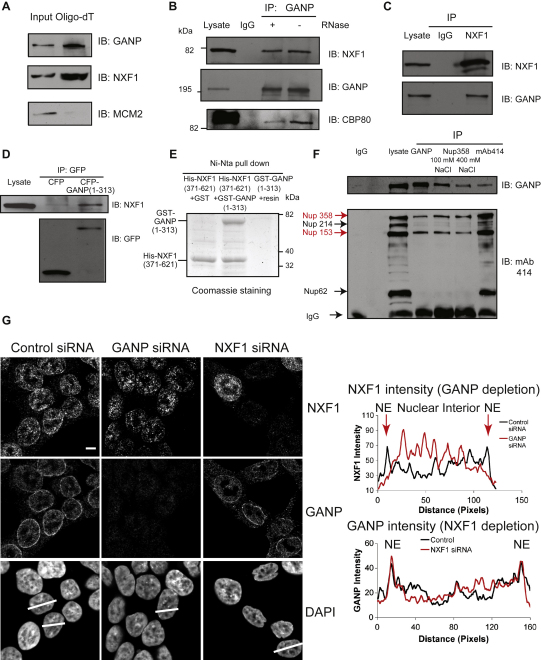
GANP Interactions with mRNA Nuclear Export Components (A) GANP interacts with nuclear poly(A)^+^RNPs. Nuclear poly(A)^+^RNA purified under nondenaturing conditions from soluble nuclear extract of HCT116 cells was analyzed by immunoblotting for associated proteins. (B) GANP interacts with NXF1 in vivo. Endogenous GANP was immunoprecipitated from nuclear extracts of HCT116 cells in the presence of RNase and blotted for GANP and NXF1. (C) NXF1 interacts with GANP in vivo. Endogenous NXF1 was immunoprecipitated from nuclear extract of HCT116 cells and blotted for GANP and NXF1. (D) GANP interacts with NXF1 through its nucleoporin homology region containing FG repeats (residues 1–313). CFP or CFP-GANP(1-313) was immunoprecipitated with GFP antibody from nuclear extracts of HCT116 cells expressing CFP or CFP-GANP(1-313) and blotted for GFP and NXF1. (E) GANP(1-313) can interact in vitro with NXF1(371-621). Recombinant GST-GANP(1-313) and His-NXF1(371-621) encoding the FG-repeat binding region of NXF1 were expressed and purified in *E. coli*. In vitro binding assays were performed with glutathione S-transferase (GST) alone or GST-GANP(1-313) incubated with His-NXF1(371-621) prebound to Ni-NTA agarose for 2 hr at 4°C. Resin was washed extensively and analyzed by SDS polyacrylamide gel electrophoresis and Coomassie staining. (F) GANP interacts with Nup153 and Nup358. Endogenous GANP was immunoprecipitated from cellular extract of HCT116 cells with anti-GANP antibody, mAb414 antibody, anti-Nup358 antibody, or sheep immunoglobin G (IgG) and blotted for GANP and mAb414. mAb414 recognizes Nup 358, 214, 153, and 62 (marked by arrows). (G) NXF1 localization is altered in GANP-depleted cells, but GANP localization is not altered in NXF1-depleted cells. Scanning analysis of NXF1 intensity in control siRNA-treated or GANP-depleted cells and also GANP intensity in control siRNA-treated or NXF1-depleted cells are shown. Nuclei used for scanning and the scanning axis are indicated by white lines. Pairs of nuclei of same scan width as determined by DAPI staining were used for scanning. Nuclear envelope (NE) and nuclear interior are indicated. Magnified view is shown in Figure S3.

To test whether GANP is associated with nuclear messenger ribonucleoproteins (mRNPs), we harvested nuclear poly(A)^+^RNPs from mammalian nuclei with oligo(dT) cellulose. Endogenous GANP specifically copurified with the poly(A) fraction, as did NXF1, a known component of nuclear mRNPs (Figure 3A). Conversely, MCM2, a component of the DNA prereplication complex, did not copurify with the poly(A) fraction (Figure 3A). These results indicate that GANP associates with nuclear mRNPs, either directly or indirectly through other proteins.

NXF1 is a major metazoan mRNA export factor [8, 9] and has a similar depletion phenotype to GANP (Figure 2D). We asked whether GANP interacts directly with NXF1 to facilitate efficient mRNA export in mammalian cells. Figures 3B and 3C show that endogenous NXF1 was coimmunoprecipitated from cell extracts by antibodies against GANP and that endogenous GANP was coimmunoprecipitated by antibodies against NXF1. NXF1 still coimmunoprecipitated with GANP in the presence of RNase A, confirming that this interaction was not RNA mediated (Figure 3B). Because NXF1 binds the FG-repeat region of Nup214 [7], which is 29% identical to the N-terminal FG motif-containing region of GANP (Figure 1A), we investigated whether this region of GANP could mediate its interaction with NXF1. Figure 3D shows that the fragment generated by the fusion of cyan fluorescent protein (CFP) to the first 313 residues of GANP did indeed interact in vivo with endogenous NXF1, but that CFP alone did not. In vitro binding assays with recombinant GANP(1-313) and the FG-repeat binding region of NXF1(371-621) [4–10] showed that this GANP fragment binds directly to NXF1 (Figure 3E), confirming that the first 313 residues of GANP are sufficient for interaction with NXF1. When overexpressed in HCT116 cells, CFP-GANP(1-313) was located throughout the nucleus, with no detectable NPC preference (Figure S3A). However, this was accompanied by an increased intensity of NXF1 staining within the nucleus coupled with a concomitant reduction at NPCs (Figure S3C) and nuclear poly(A)^+^RNA accumulation in a pattern identical to that of CFP-GANP(1-313) (Figure S3D). This effect was not due to increased NXF1 expression following CFP-GANP(1-313) expression (Figure S3B). These results suggest that the N-terminal GANP(1-313) fragment retained NXF1 and its bound mRNA within the nucleus, inhibiting their recruitment to NPCs and export (Figure S3).

The observations that GANP interacts directly with NXF1, that it is present in both the nuclear envelope and the nuclear interior, and that it is required for efficient export of poly(A)^+^RNA from the nucleus suggest a role for GANP in mammalian mRNA nuclear export whereby GANP recruits NXF1-containing mRNPs in the nuclear interior and delivers them to the NPC. One prediction of this hypothesis is that GANP depletion should reduce the association of NXF1 with NPCs. Figure 3G shows that GANP depletion indeed decreased the amount of NXF1 at the NPC, with a corresponding increase in the nuclear interior, whereas the distribution of GANP was unaltered by NXF1 depletion. These results suggest that GANP contributes to the recruitment of NXF1 from the nuclear interior to NPCs but that GANP recruitment to the NPC is independent of NXF1. However, because NXF1 can itself bind directly to NPCs through transient interactions with FG nucleoporins [4, 5, 7, 8, 25, 27–29], GANP may contribute to the efficient NPC recruitment of NXF1.

**Figure 4:**
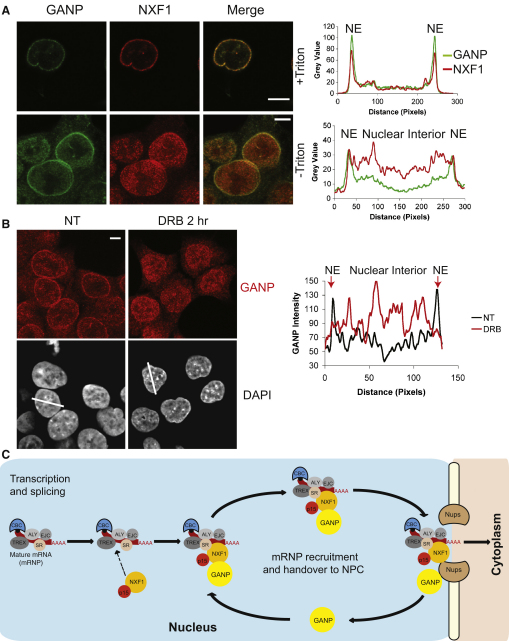
Dynamic Role of GANP in the Mammalian mRNA Export Pathway (A) GANP colocalizes with NXF1. Immunofluorescence of HCT116 cells with anti-GANP and anti-NXF1, respectively, is shown. Scale bar represents 5 μm. HCT116 cells were also treated with Triton X-100 prior to fixation to remove soluble material. Scanning analysis of GANP or NXF1 intensity is also shown. Nuclear envelope (NE) and nuclear interior are indicated. (B) Treatment with transcription inhibitor DRB redistributes GANP into foci throughout the nucleus, with concomitant reduction in NPC staining. HCT116 cells were treated with RNA Pol II-specific transcription inhibitor DRB for 2 hr, and immunofluorescence was performed with anti-GANP and anti-NXF1, respectively. Scanning analysis of GANP intensity in untreated or DRB-treated HCT116 cells is also shown with the scanning axes indicated by white lines. Pairs of nuclei of same scan width were used for scanning. Nuclear envelope (NE) and nuclear interior are indicated. (C) A model of the role of GANP in mammalian mRNA export. We propose that GANP is recruited to messenger ribonucleoproteins (mRNPs) after NXF1 is bound and that it acts as a courier to collect and deliver these mRNPs to the NPC. At the NPC, FG nucleoporins displace GANP from the mRNP, freeing mRNP to pass through the NPC, facilitated by interactions between NXF1 and the FG repeats that line the channel.

The proportion of GANP located at NPCs was higher than that seen for NXF1 (Figure 4A), consistent with GANP's contributing to the NPC recruitment of NXF1, but not vice versa (Figure 3G), and suggesting that GANP binds to NPCs. Consistent with this hypothesis, GANP coimmunoprecipitated with a fraction containing Nup153 and Nup358 but not Nup62 (Figure 3F), and also with either mAb414 or Nup358 antibodies (Figure 3F). These results indicate that the proportion of GANP localized to NPCs interacts either directly or indirectly with a subset of nucleoporins. However, it is unlikely that GANP is an integral NPC protein, because GANP was not identified in a large-scale screen for mammalian NPC components [30] and a proportion of GANP is inside the nucleus. Therefore, GANP interacts with both NXF1 and NPCs, supporting the hypothesis that it functions at a late stage in mRNA export.

Nuclear mRNP export is a dynamic process, and most actively transcribed genes localize at focal concentrations of RNA polymerase II (RNA Pol II) deep in the nucleus called transcription factories [18]. We have shown that a proportion of GANP was localized in nuclear foci and that depletion of GANP resulted in retention of mRNPs in punctate nuclear foci. The role for GANP in mRNA export indicated by our data suggests that GANP is actively involved in recruitment and transport of mRNPs through the nucleus to NPCs. If GANP is shuttling and carrying mRNPs from sites of transcription and processing to NPCs, then inhibition of transcription should perturb the distribution of GANP along this pathway. Therefore, we examined the effect of inhibiting transcription on the localization of GANP. In untreated intact cells, GANP was concentrated at the NPC, with a lower concentration in the nucleus (Figure 4B). Strikingly, treatment with DRB, a specific inhibitor of RNA Pol II elongation [31], redistributed GANP into foci throughout the nucleus, with less at the NPC (Figure 4B). A similar result was observed following treatment with the more general transcription inhibitor actinomycin D (Figure S4). These results suggest that GANP is mobile and that it shuttles between transcription factories and the NPC.

Our results are consistent with a model for GANP function in mammalian mRNA nuclear export whereby GANP is recruited to mature mRNPs after NXF1 is attached, enhancing the delivery of mRNPs to the NPC (Figure 4C). In contrast to yeast, where the GANP homolog Sac3 functions with Sus1 and Cdc31 in the TREX-2 complex to target active genes such as *GAL1* to NPCs [8, 17, 36], we propose that in mammalian cells, GANP facilitates targeting of NXF1-containing mRNPs to NPCs. We propose that when these mRNPs reach NPCs, the higher concentrations of FG motifs in FG nucleoporins displace GANP from NXF1 in the mRNP, freeing it to pass through the pores, facilitated by interactions between NXF1 and the FG repeats that line the transport channel. This hypothesis is supported by several lines of evidence: (1) GANP associates with nuclear mRNPs, and treatment with transcription inhibitors redistributes GANP in foci throughout the nucleus, with a reduction at NPCs, consistent with a shuttling function between transcription factories and the NPC; (2) GANP interacts with the same domain of NXF1 that interacts with FG-repeat nucleoporins; (3) GANP depletion results in retention of mRNPs in nuclear foci and not at NPCs, similar to the pattern seen following NXF1 depletion but not that observed following Nup depletion; (4) overexpression of the GANP(1-313) fragment that localizes in the nucleus and not at NPCs retains NXF1 within the nucleus, preventing its efficient recruitment to NPCs and subsequent nuclear export of NXF1-bound mRNPs; and (5) GANP depletion reduces the amount of NXF1 at NPCs with a corresponding increase in the nuclear interior, suggesting that GANP contributes to the recruitment of NXF1 from the nuclear interior to NPCs.

Our data establish a role for GANP distinct from those proposed previously [19, 20, 32–35], which focused on cells of the immune system in which GANP was discovered but failed to account for GANP's being expressed universally rather than only in cells of the immune system. By contrast, our data instead suggest that other phenotypic effects of GANP depletion could be secondary consequences of the function of GANP in mRNA export.

## Supplemental Information

Document S1. Supplemental Experimental Procedures and Four Figures
