# Supplementary material for: mRNA Export from Mammalian Cell Nuclei Is Dependent on GANP
Source: Curr Biol. 2010 Jan 12;20(1):25–31. doi: 10.1016/j.cub.2009.10.078 (PMC2869303; doi:10.1016/j.cub.2009.10.078)
Supplement: Document S1. Supplemental Experimental Procedures and Four Figures [file mmc1.pdf]

Current Biology, Volume *20*

Supplemental Information

mRNA Export from Mammalian Cell Nuclei Is Dependent on GANP

Vihandha O. Wickramasinghe, Paul I.A. McMurtrie, Anthony D. Mills, Yoshinori Takei, Sue Penrhyn-Lowe, Yoko Amagase, Sarah Main, Jackie Marr, Murray Stewart, and Ronald A. Laskey

Supplemental Experimental Procedures

Immunofluorescence and cell treatments

Cells were fixed in 2 or 4 % paraformaldehyde, and permeabilised in PBS, 0.1 % Triton X-100 (Sigma), 0.02 % SDS for 10 mins at room temperature. After 30 mins blocking (same buffer + 1 % BSA), coverslips were incubated with the appropriate primary and secondary antibodies (Molecular Probes) and examined using a Zeiss LSM510 Meta confocal microscope. For some experiments, cells were permeabilised prior to fixation with PBS, 0.1 % Triton X-100 for 5 mins on ice. For some experiments, cells were treated with Actinomycin D (5 μg/ml) or DRB (50 μg/ml) (Sigma) for between 2 to 4 hours. For antibody access experiments, cells were permeabilised after fixation with 0.004 % digitonin (ICN Biomedicals) for 5 mins on ice, or with 0.2 % Triton X-100 for 5 mins at room temperature. Scanning analysis of cells was performed using ImageJ software (NIH). All images used for comparative analysis were acquired using identical microscope settings. A line width of 20 was used, and pairs of cells with nuclei of same scan width as indicated by DAPI staining were used for analysis. All analyses are representative of the cell population.

Constructs and Transfections

For cloning of pECFP-GANP(1-313), the GANP fragment was excised from pCRBlunt-GANP using BamH1 and ligated to pECFP-C1 (BD Biosciences) via BamH1.

Transfections with plasmid DNA where indicated were carried out with Genejuice as per manufacturers instructions (Novagen).

siRNA mediated depletion

For siRNA mediated depletion, 10^6 HCT116 cells were transfected (Amaxa AG) with 10 μl of 20 μM siRNA (GANP siRNA1-GAGAGGACCUAAGUCAAUA; GANP siRNA2- AGCUUGCAGUGGUACAUUU; NXF1 and Nup153 siRNA- GenomeWide siRNA (Qiagen)). Cells were harvested either 48 or 72 hours post-transfection. Efficiency of depletion was monitored by immunoblotting with the indicated antibodies. A control siRNA (GAGAGGUCCAAAGUCAAUA) identical to GANP siRNA1 but for 2 bases was used for all experiments.

Immunoprecipitations and Antibodies

For nucleoporin immunoprecipitations, 5 x10^6 cells were lysed in 50 mM HEPES pH 7.4, 300 mM NaCl, 0.5% NP-40, 10 mM EDTA, 2 mM DTT and protease inhibitors (Roche) for 15 mins on ice, and lysate passed through a 21 G syringe. Following centrifugation, lysate was pre-cleared with Protein A Sepharose (Amersham) and incubated with 2-5 μg of indicated primary antibody for 2 hours. Samples were incubated with 50 μl of Protein A Sepharose (GE Healthcare) overnight with rotation, washed with lysis buffer 4 times, then eluted with 50 μl SDS loading buffer. Antibodies used were anti-GANP (raised against recombinant GANP 1050-1250aa purified in *E.coli*), mAb414 (Babco), NXF1 (Abcam), Nup358 (Abcam), CBP80 (Abcam), STAT2 (Cell Signaling), Lamin B (Calbiochem), β-actin (Abcam) and GFP, which recognises CFP moiety (Clontech). For NXF1, GANP and CFP-GANP(1-313) immunoprecipitations, nuclear extract was used. Briefly, 10 x 10^6 cells were permeabilised with 10 mls of 50 μg/ml digitonin (ICN Biomedicals), 100 mM NaCl, 10 mM Tris pH 8.0 and protease inhibitors (Roche) for 10 mins on ice. Following centrifugation at 800 g for 3 mins, pelleted nuclei were washed with 10 mM Tris pH 8.0, 100 mM NaCl, protease inhibitors and nuclei were lysed with 400 μl of 10 mM Tris pH 8.0, 500 mM NaCl, 0.1 % NP-40 and protease inhibitors for 10

mins on ice. Following centrifugation at 14000 rpm for 30 mins at 4^0C, supernatant was diluted in lysis buffer without NaCl so that final concentration of NaCl was 150 mM. For some samples, RNase (0.2 mg/ml) was added to nuclei lysis buffer and all subsequent buffers. Samples were then processed as above.

Protein expression, purification and in *vitro* binding assays

GST-GANP(1-313) and His-NXF1(371-621) were expressed individually in BL21 cells. Cultures were grown at 37^0C to an OD_{600} of 0.6, then induced by addition of IPTG to 1mM and grown at 30 or 37^0C for 4 hours. Cells expressing GST-GANP(1-313) were lysed in 1xPBS, protease inhibitors (Roche), and sonicated on ice. 1 % Triton X-100 (Sigma) was added and incubated for 30 mins at 4^0C. GANP fragment was bound to Glutathione Sepharose (GE Healthcare) for 2 hours at 4^0C with rotation, and beads washed extensively to remove unbound proteins. GST-GANP(1-313) was eluted with 50 mM Tris-HCl pH 8.0, 20 mM reduced glutathione. Cells expressing His-NXF1(371-621) were lysed in lysis buffer (50 mM phosphate buffer, 300 mM NaCl, 10 mM imidazole, pH 8.0), and lysozyme added for 30 mins at 4^0C. After sonication on ice, lysate was centrifuged at 10 000 g for 25 mins at 4^0C. Cleared lysate was purified over Ni-Nta agarose (Qiagen) and washed extensively with lysis buffer with 20 mM imidazole, followed by lysis buffer with 40 mM imidazole. Binding assays were performed by immobilising His-NXF1(371-621) on Ni-Nta agarose resin. Purified GST or GST-GANP(1-313) was incubated with resin for 2hrs at 4^0C and washed extensively with 50 mM phosphate buffer pH 8.0, 150 mM NaCl, 10 mM imidazole. Samples were analysed by SDS-PAGE.

RNA FISH

RNA FISH was performed as previously described [1] using an oligo(dT) primer (Sigma). Nuclear accumulation of poly(A)+ RNA in GANP depleted cells was quantitated and compared to cells transfected with the control siRNA. At least 1400 nuclei per sample were analysed using ScanR acquisition and analysis software

(Olympus). Nuclear Cy3 intensity and standard deviation were calculated for each coverslip. Nuclear Cy3 intensity was measured for the whole nucleus and not adjusted for the unstained nucleoli.

Poly(A)+RNA isolation

1.5 x 10^7 HCT116 cells were harvested for nuclear poly(A)+RNA IP essentially as described [2], except 0.2 U/µl of RNase OUT (Invitrogen) was used as RNase inhibitor. Also, nuclei were obtained by digitonin permeabilisation as above, and nuclei were lysed as above to obtain soluble nuclear extract for IP. Samples were analysed by SDS-PAGE followed by immunoblotting with anti-NXF1 (Abcam) as positive control, anti-GANP, and anti-MCM2 (BD Biosciences) as negative control.

Supplemental References

1. Herold, A., Klymenko, T., and Izaurralde, E. (2001). NXF1/p15 heterodimers are essential for mRNA nuclear export in Drosophila. RNA *7*, 1768-1780.
2. Kozlova, N., Braga, J., Lundgren, J., Rino, J., Young, P., Carmo-Fonseca, M., and Visa, N. (2006). Studies on the role of NonA in mRNA biogenesis. Exp Cell Res *312*, 2619-2630.

A

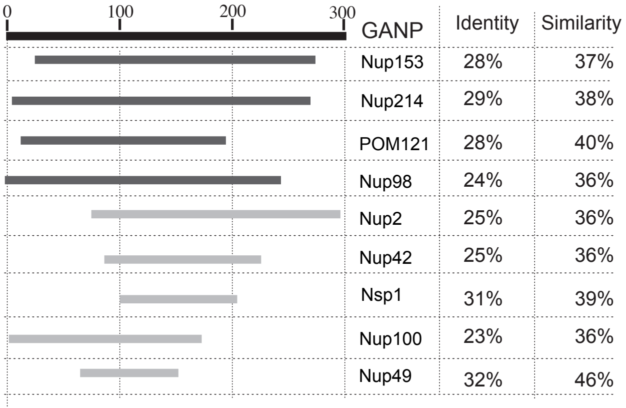

B

Alignment (aa)	Identity (%)	Similarity (%)
GANP (634-990) Xmas-2 (201-560)	43	53
GANP (634-990) Sac3p (196-561)	25	36
Xmas-2 (201-560) Sac3p (196-561)	23	36
GANP (634-990) Xmas-2 (201-560) Sac3p (196-561)	53 (2 out of 3)	67 (2 out of 3)

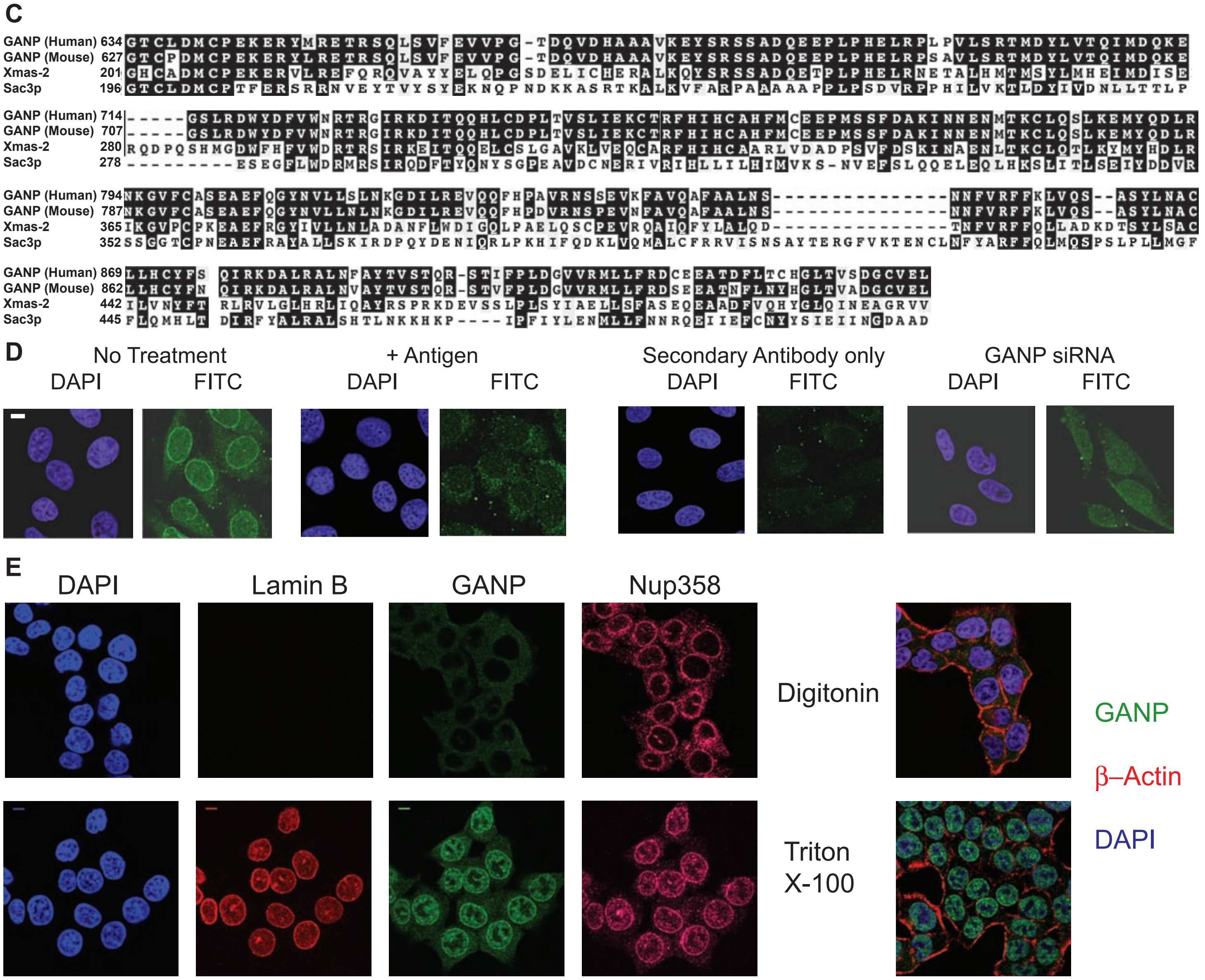

Figure S1- GANP contains regions of homology to nucleoporins and mRNA exporters and is localised to the nuclear face of the NPC. (A) GANP sequence alignment (aa 1-300) with nucleoporins. Bars represent approximate regions of homology with GANP. Identity and similarity between respective protein sequences of nucleoporins and GANP are indicated. Dark grey bars represent Homo sapiens proteins, light grey represent S.cerevisiae proteins. (B) and (C) Table representing identity (%) and similarity (%) between protein sequences of a region of GANP (Human, 634-936, and mouse), Sac3p (*S.cerevisiae*) and Xmas-2 (*Drosophila*) based on GANP protein alignment with Sac3p and Xmas-2 using ClustalW and Boxshade. Black boxes represent identity, grey boxes represent similarity. (D) Characterisation and validation of GANP antibody. Anti-GANP immunofluorescence was carried out on U20S cells with or without antigen and also on GANP depleted cells. Secondary antibody only control is shown. Nuclei were stained with DAPI. (E) GANP is localised to the nuclear face of the NPC. HCT116 cells were permeabilised with either digitonin, which preferentially permeabilises the plasma membrane, or Triton X-100, which permeabilises both plasma and nuclear membranes, and stained with anti-GANP, Lamin B and Nup358 antibodies. The integrity of the nuclear and plasma membranes following digitonin or Triton X-100 permeabilisation is confirmed by staining of Nup358, a nucleoporin that is localised to the cytoplasmic face of the NPC, of Lamin B, an inner nuclear membrane protein and of β–actin, a cytoplasmic protein.

A

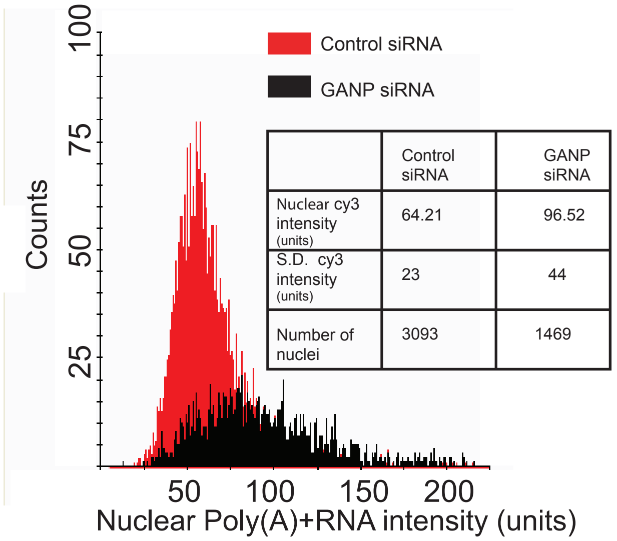

	Control siRNA	GANP siRNA
Nuclear cy3 intensity (units)	64.21	96.52
S.D. cy3 intensity (units)	23	44
Number of nuclei	3093	1469

B

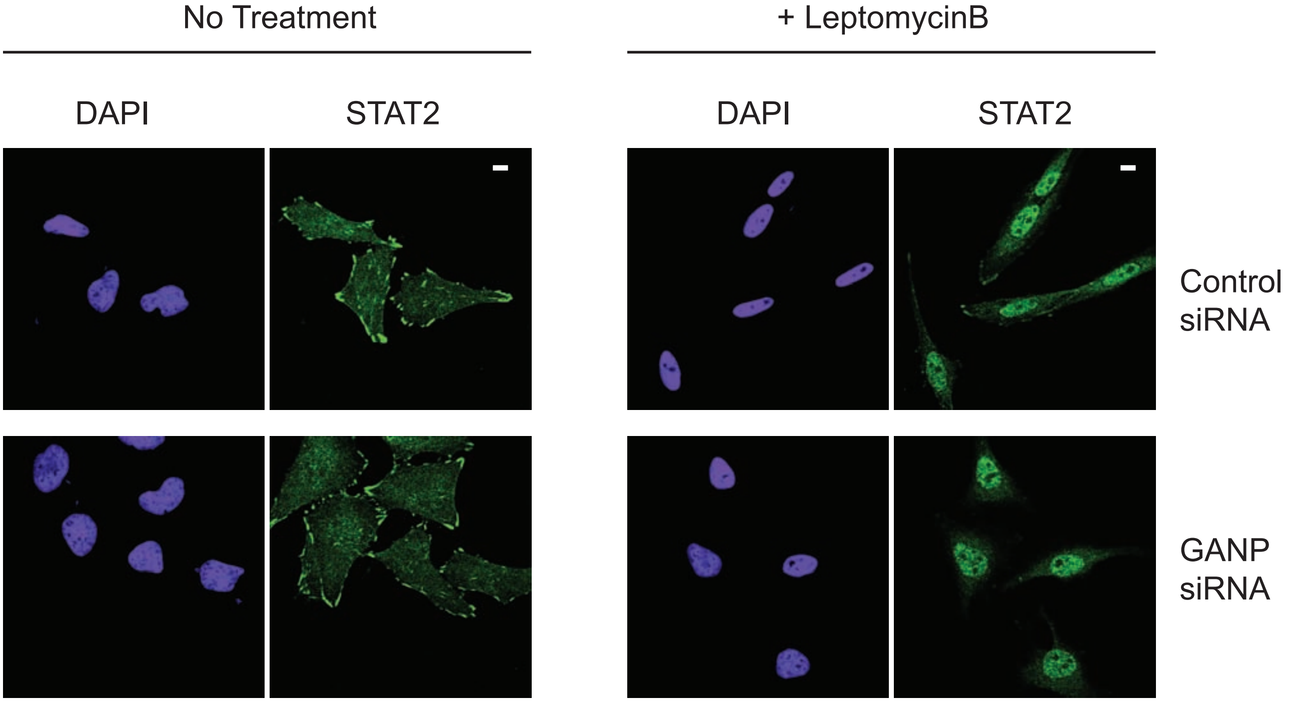

Figure S2- Quantitation of Nuclear poly(A)+RNA intensity in GANP depleted cells and NPC is functional in GANP depleted cells. (A) Quantitation of results shown in Figure 2A. Coverslips were analysed for nuclear Cy3 intensity using ScanR acquisition and analysis software (Olympus). Nuclear Cy3 intensity was measured for the whole nucleus and not adjusted for the unstained nucleoli. A plot of counts vs. nuclear Cy3 intensity is shown. Black represents GANP siRNA treated cells, and red represents control siRNA treated cells. Nuclear Cy3 intensity and standard deviation are shown for each population in the table. For ScanR analysis, cells were synchronised in S-phase prior to harvest. At least 1400 nuclei were counted for each coverslip. (B) NPC is functional in GANP depleted cells. Nuclear import and CRM-1 dependent export of endogenous STAT2 proceed in the absence of GANP. Anti-STAT2 immunofluorescence was carried out on HeLa cells transfected with control siRNA or GANP siRNA and treated with leptomycin B (20 nM) for 1 hour to inhibit CRM-1 dependent export.

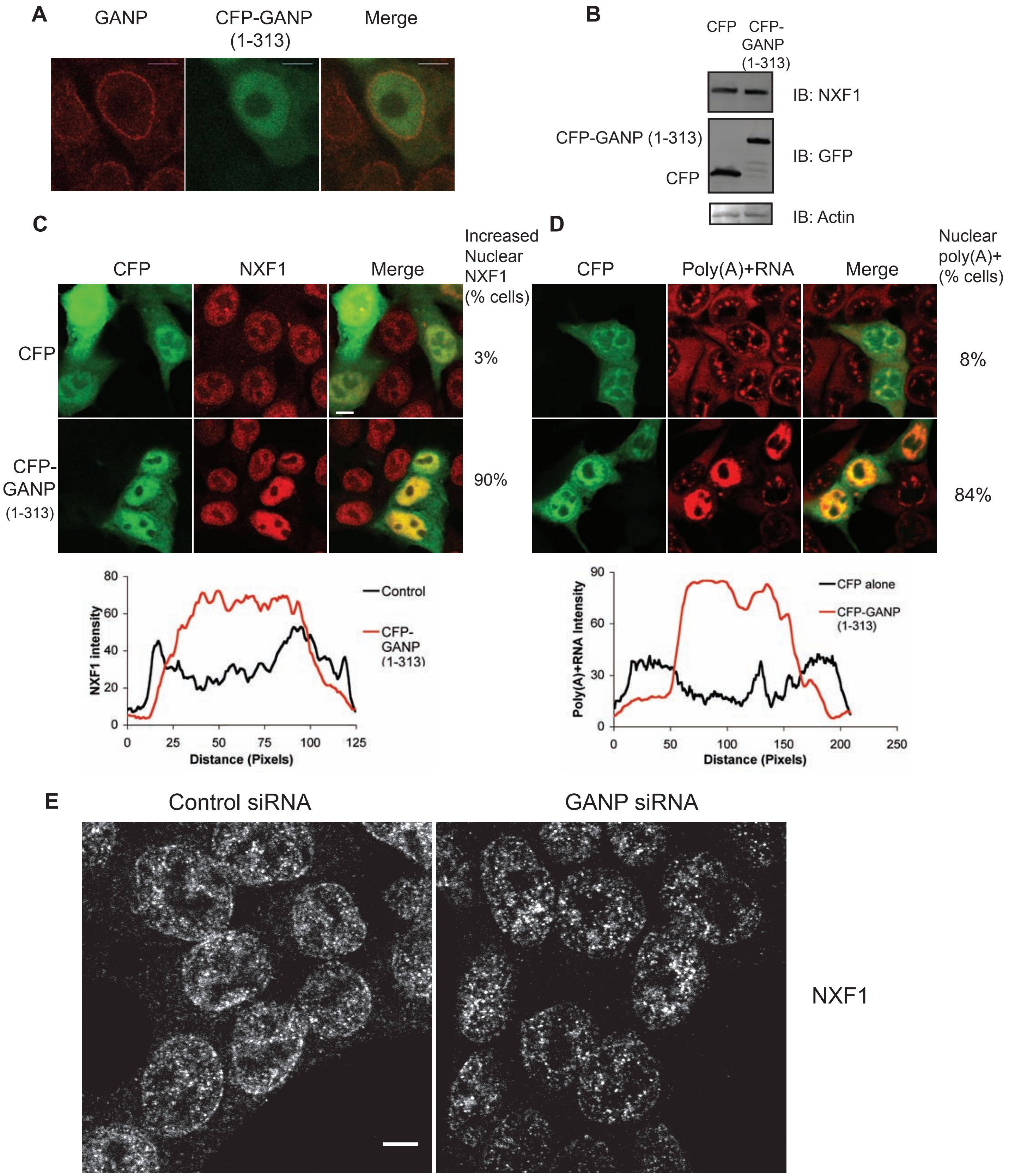

Figure S3- Expression of CFP-GANP(1-313) causes nuclear accumulation of NXF1 and poly(A)+RNA. (A) Endogenous GANP localisation is unchanged in HCT116 cells overexpressing CFP-GANP(1-313). Anti-GANP immunofluorescence was carried out on HCT116 cells overexpressing CFP-GANP(1-313). Note that the anti-GANP antibody was raised against a different epitope (aa1050-1250) and hence does not recognise GANP(1-313). (B) Expression of CFP-GANP(1-313) does not affect NXF1 expression level. HCT116 cells expressing CFP or CFP-GANP(1-313) were analysed for total NXF1 levels by western blotting with an antibody against NXF1. (C) Expression of CFP-GANP(1-313) causes nuclear accumulation of endogenous NXF1. Immunofluorescence was performed on HCT116 cells 48 hours following transfection with vector expressing CFP alone or CFP-GANP(1-313) using an anti-NXF1 antibody. Percentages of CFP or CFP-GANP(1-313) expressing cells displaying increased nuclear NXF1 staining are indicated. At least 250 cells were counted. Scanning analysis of NXF1 intensity is also shown. (D) Expression of CFP-GANP(1-313) causes nuclear accumulation of poly(A)+RNA. HCT116 cells were analysed by RNA FISH 48 hours post-transfection as before. Scanning analysis of poly(A)+RNA intensity is shown. (E) Magnified view of Figure 3G is shown.

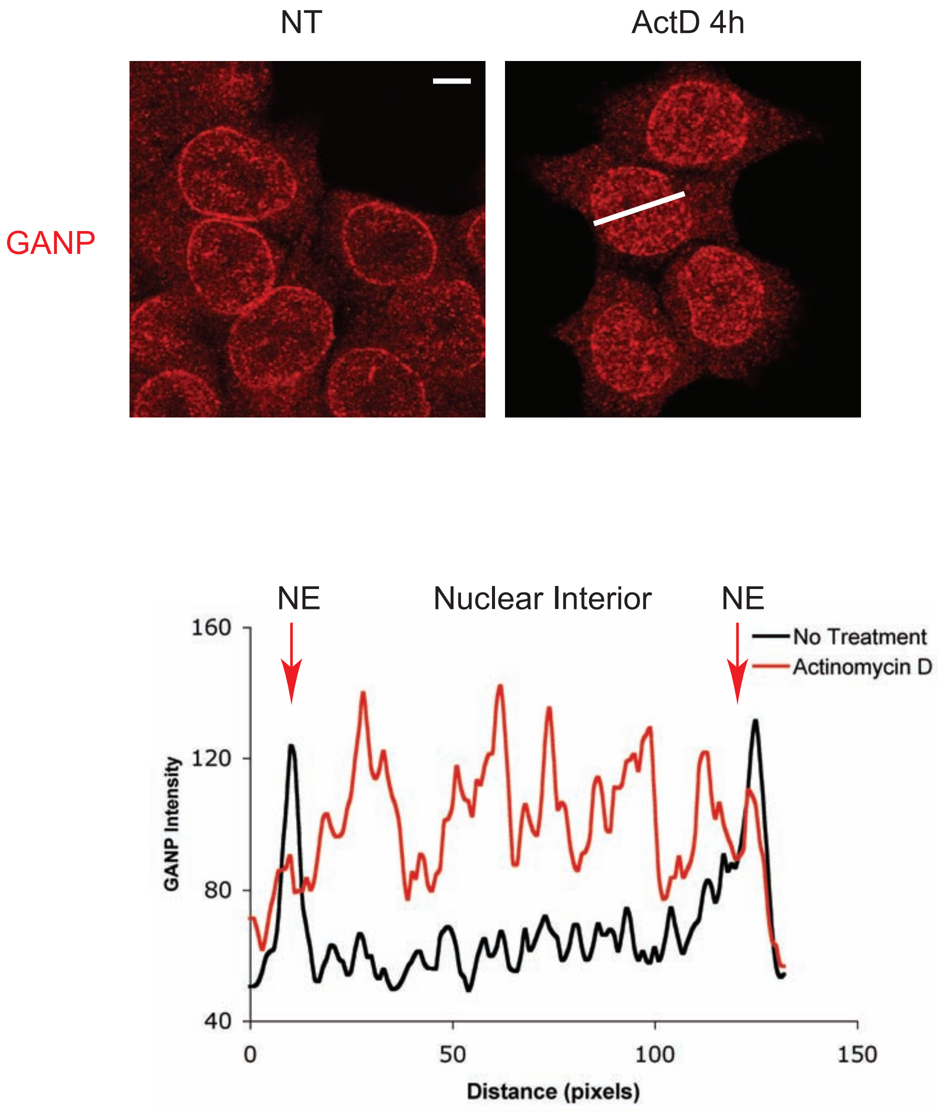

Figure S4- GANP localisation is altered following treatment with Actinomycin D
HCT116 cells were treated with transcription inhibitor Actinomycin D for 4 hours and immunofluorescence was performed using anti-GANP and anti-NXF1 antibodies respectively. Scanning analysis of GANP intensity in untreated or Actinomycin D treated HCT116 cells using ImageJ software was performed. Nuclear envelope (NE) and nuclear interior are indicated.
